# Association between maternal exposure to housing renovation and offspring with congenital heart disease: a multi-hospital case–control study

**DOI:** 10.1186/1476-069X-12-25

**Published:** 2013-03-25

**Authors:** Zhen Liu, Xiaohong Li, Nana Li, Shengli Li, Kui Deng, Yuan Lin, Xinlin Chen, Fengzhi You, Jun Li, Dezhi Mu, Yanping Wang, Jun Zhu

**Affiliations:** 1 National Center for Birth Defect Monitoring, West China Second University Hospital, Sichuan University, Sec.3 No.17, South RenMin Road, Chengdu, Sichuan, China; 2 Laboratory of Molecular Epidemiology for birth defect, West China Institute of Women and Children's Health, Sichuan University, Chengdu, China; 3 Department of Ultrasound, Shenzhen Maternity & Child Healthcare Hospital, Affiliated to Southern Medical University, Shenzhen, China; 4 Department of Obstetrics & Gynecology, Fujian provincial Maternal and Child Healthcare Hospital, Fuzhou, China; 5 Department of Ultrasound, Hubei provincial Maternal and Child Healthcare Hospital, Wuhan, China; 6 Department of women sanitation, Henan provincial Maternal and Child Healthcare Hospital, Zhengzhou, China; 7 Department of Ultrasonic Diagnosis, Xijing Hospital, Fourth Military Medical University, Xi’an, China; 8 Department of Pediatric, West China Second University Hospital, Sichuan University, Chengdu, China

**Keywords:** Air pollution, Congenital heart disease, Pregnancy, Reproductive health, Housing, Indoor air

## Abstract

**Background:**

Congenital heart disease (CHD) is one of the most prevalent birth defects. Housing renovations are a newly recognized source of indoor environmental pollution that is detrimental to health. A growing body of research suggests that maternal occupational exposure to renovation materials may be associated with an increased risk of giving birth to fetuses with CHD. However, the effect of indoor housing renovation exposure on CHD occurrence has not been reported.

**Methods:**

A multi-hospital case–control study was designed to investigate the association between maternal periconceptional housing renovation exposure and the risk of CHD for offspring. In total, 346 cases and 408 controls were enrolled in this study from four hospitals in China. Exposure information was based on a questionnaire given to women during pregnancy. The association between housing renovation exposure and CHD occurrence was assessed by estimating odds ratios (OR) with logistic regression models adjusted for potential confounders.

**Results:**

The risk for CHD in offspring was significantly associated with maternal exposure to housing renovations (AOR: 1.89, 95% CI: 1.29-2.77). There were similar risks for cardiac defects with or without extra-cardiac malformation (AOR of 2.65 and 1.76, respectively). Maternal housing renovation exposure may increase the fetus’ risk of suffering from conotruncal defect or anomalous venous return. There were significant risks for cardiac defects if the pregnant woman moved into a new house within one month after decoration at either 3 months before pregnancy (AOR: 2.38, 95% CI: 1.03 to 5.48) or during first trimester (AOR: 4.00, 95% CI: 1.62 to 9.86).

**Conclusions:**

Maternal exposure to housing renovations may have an increased risk of giving birth to fetuses with some selected types of CHD. This relationship was stronger for women who moved into a newly decorated house. However, considering the limited number of subjects and the problem of multiple exposures, more research is needed to clarify the effects seen here.

## Background

Congenital heart disease (CHD) is the most prevalent type of recognized structural birth defect among newborns. Worldwide, approximately 6 to 10 ‰ of live births suffer from a CHD [1,2]. The prevalence of CHD increases up to 53-79 ‰ when including trivial lesions, abortions and stillbirths [3,4]. All cardiac defects accounts for a high proportion (46%) of all infant deaths caused by congenital malformations [5]. Although certain genetic conditions and some environmental factors are found to be linked with the occurrence of CHD, the etiology of most nonsyndromic CHD is still unknown [6,7].

Like most developing countries, housing conditions in China have continually improved with the progression of living standards. An increasing number of people have moved into newer houses, while many families have chosen to renovate the old homes instead. Then, most houses need to be decorated before moving in. However, some inferior decoration materials may inevitably release a large amount of hazardous substances, such as organic solvents, volatile organic compounds (VOCs), formaldehyde and heavy metals [8,9]. Therefore, housing renovation has become a new source of indoor environmental pollution.

A growing body of research has found that prenatal exposure to ambient air pollution may be associated with adverse birth outcomes [10-12]. As one type of indoor air pollution, housing renovations have been shown as a threat to health, especially for fetuses and children. More evidence has revealed that when women were exposed to indoor renovations during pregnancy, infants were prone asthma, eczema and allergies in early childhood [13-17]. However, very few studies have reported the association between indoor renovation and congenital anomalies, such as CHD.

A few studies have found that maternal occupational exposure to renovation materials, such as paints, dyes and glues may be a risk factor for CHD in offspring [18,19]. In addition, a Danish National Birth Cohort (DNBC) study indicated that non-occupational exposure to paint fumes may be associated with congenital anomalies in a general population in the home environments [20]. However, this connection has not been observed with home exposure to renovation and the occurrence of CHD. Since people spend nearly 80% of their time indoors every day, the quality of indoor air has a direct impact on people's health. Therefore, it is necessary to provide insight into the specific effects of maternal indoor pollution exposure on the risk for developing birth defects, such as CHD.

In 2009, we implemented a program to study gene-environment factors related to CHD occurrence in China. The program was designed as a hospital-based case–control study. The data in the present study were derived from the epidemiologic databank of this program. The study focused on the potential effect of maternal exposure to housing renovations on the risk of CHD in offspring, as well as, assessed the critical exposure window.

## Methods

### Study population and inclusion criteria

Participants were recruited during prenatal care visits from Feb. 2010 to Oct. 2011 in four tertiary maternal and child hospitals (Guangdong, Fujian, Henan and Hubei provinces) in China. The four participating hospitals were qualified as regional prenatal diagnosis centers that provided genetic screening and diagnostic testing for fetal defects with high level ultrasound technology [21,22].

CHD was defined as “a gross structural abnormality of the heart or intrathoracic great vessels that is actually or possibly of functional significance”, as described by Mitchell [23]. The inclusion criteria for cases included the following: (1) singleton pregnancies, (2) greater than 14 weeks for gestational age, (3) fetuses that were diagnosed with a defined CHD, and (4) all fetal heart defects and malformations that were confirmed after birth or abortion. The cases contained live birth, stillbirth and abortions with any CHD. The controls were recruited from the same hospital during the same study period as the cases. We selected the first voluntary and qualified pregnant woman as a control just after one case was recruited. The inclusion criteria for controls included the following: (1) singleton pregnancies, (2) no more than a two week difference in gestational age when matched with the case, and (3) fetuses that were not diagnosed with a CHD or other congenital malformations. All participants were administered the same questionnaire in the same way by one assigned investigator. Case and control fetuses with unclear diagnoses or confirmed as having chromosomal abnormalities or syndromes by cytogenesis analysis were all ruled out. Participants who had an explicit history of occupational exposure to organic solvents or engaged in the production or sale of decoration materials were excluded. Cases and controls with a family history of CHD or gestational diabetes were also removed.

### Subject enrollment and data collection

The ethical approval of this project was authorized by Sichuan University Ethics Committee (No.2010004). All cases and controls were checked by prenatal systematic echocardiography when pregnant women received a prenatal examination at the designated hospital. Those who were in strict accordance with the case or control inclusion criteria were invited to join in our project. A face-to-face questionnaire interview was conducted after the pregnant woman fully understood the program and signed consent forms. All cases and controls were followed for three months after delivery to ascertain the disease. The questionnaire, ultrasound data (including static and dynamic images), and clinical examination results were sent to the project team for review.

### Determination and classification of cardiac defects

The final diagnosis for each case of live birth was confirmed within the first week after delivery through routine examination, heart auscultation and neonatal echocardiography by pediatric cardiologists. Furthermore, all static and dynamic echocardiography images of cases of CHD were reviewed by at least 4–5 national-level prenatal ultrasound specialists and pediatric cardiologists to ensure the accuracy of the final diagnosis. Cases of stillbirths and terminated pregnancies were confirmed by pathological autopsy for the final diagnosis.

CHD cases were divided into two groups, namely “isolated” and “complex” group. Isolated cardiac defects were considered as abnormalities with only cardiac malformation but without any other form of diagnosed noncardiac malformations. CHD cases that were associated with other congenital extra-cardiac defects were considered as complex malformations.

Meanwhile, all cardiac defects cases were classified into six subtypes based on the anatomic lesion: (i) septal, including atrial septal defects, ventricular septal defects (VSD), and endocardial cushion defect; (ii) conotruncal, including transposition of great arteries (TGA), tetralogy of Fallot, truncus arteriosus, and double outlet right ventricle; (iii) right-sided obstructive, including pulmonary valve stenosis, pulmonary atresia, tricuspid atresia, and Ebstein anomaly; (iv) left-sided obstructive, including aortic valve stenosis, hypoplastic left heart syndrome and variants, coarctation of the aorta, and interrupted aortic arch, (v) anomalous venous return, including total and partial anomalous pulmonary or systematic venous return; and (vi) others, including single ventricle, heterotaxias, and other cardiac structural abnormalities.

### Exposure assessment

The questionnaire was formulated according to literature reviews and expert opinions regarding the environmental factors exposure during the periconceptional period. Information on exposure to housing renovations was collected through specific questions, including the time of maternal exposure to indoor renovations. Time periods included (i) 7–12 months before pregnancy, (ii) 4–6 months before pregnancy, (iii) 0–3 months before pregnancy, and (iv) the first trimester. The mother was also asked how long it took her to move into the new house after renovation and at what exact time the woman occupied the renovated house.

Housing renovation is defined as the transformation of the interior by the use of at least one or more of the following types of materials: marbles, plywood, laminated board, carpets, ceramic tile, oil-based paint, latex or acrylic coating and wallpapers. The following situations were not counted as renovations: (i) renovation that did not occur before moving into a new house; (ii) renovation that occurred over a year before conception or after the second trimester; (iii) never having moved into the renovated house to live; (iv) only having bought new furniture or adornments, such as beds, wardrobes, sofas, tables, chandeliers, etc., and anything that does not coincide with the above conditions.

### Potential confounders

Referring to the literature, potential confounders are those factors that correlate with both the main determinant (renovation) and outcome (CHD). Information on potential confounders was obtained on sociodemographic factors including maternal age (at the time of the last menstrual period), maternal education level (primary school or less, junior school, senior high school, and college or advanced degree), maternal residence area (urban, suburban or rural), and health status from three months preconception to first trimester, including use of folic acid supplements (yes or no), and maternal acute or chronic respiratory diseases (yes or no).

Additionally, more factors were investigated as covariates, including exposure to a factory or landfill nearby (<1000 meters, yes or no), cooking at home (yes or no), air ventilation (good, average or poor), and maternal smoking or environmental tobacco smoke (ETS) exposure (paternal smoker and/or other nearby smokers, yes or no).

### Statistical analysis

A case–control analysis was performed to assess the potential effect using the database of identified cases and controls. The statistical calculations were performed using software SPSS, version 16.0.0 (IBM, 1989–2007; TEAM EQX). The composition ratio of potential factors was calculated first. Differences in proportions between cases and controls were calculated using *t*-test and *Chi-square* test (two-tailed values of *P* < 0.05).

The associations between renovations and different types of CHDs were assessed by calculating crude odds ratios (CORs) using univariate logistic regression analyses. In subsequent models that included the potential confounding variables, the adjusted odds ratios (AORs) were calculated using multivariate logistic regression. 95% Confidence Intervals (95%CIs), excluding 1.000, were considered to be statistically significant. All confounder factors were included based on change of main effect. We select covariate factors on the basis of the results of the bivariate analysis firstly. The remaining variables were successively incorporated according to the likelihood ratio of the changes (p < 0.05) in the model. Maternal age, maternal education level, place of residence, folic acid intake, ETS, factory or landfill nearby and air ventilation were retained as covariates. Because the distribution of participants among hospitals was uneven, this term was also included in the model.

## Results

From February 2010 through October 2011, 560 women who had conceived fetuses with CHD and 472 women who had conceived fetuses without any birth defects were enrolled in the study. Of these participants, 183 cases were diagnosed as having a chromosomal abnormality, a nonchromosomal syndrome or had an ambiguous diagnosis; these cases were excluded from further analysis. In addition, 50 control mothers were removed because the follow-ups were not able to be conducted or anomalies in the performance of the following-up. In the two groups, there were 14 case and 6 control mothers with occupational exposure, 12 cases and 4 controls who had a family history of CHD, and 5 case and 4 control mothers with gestational diabetes, all of these individuals were excluded from this study. Ultimately, 346 cases and 408 controls were used for subsequent analysis.

### The analysis of characteristics between the case and control groups

Many characteristics were significantly different between case and control mothers, except for maternal cooking and maternal acute or chronic respiratory disease at three months before pregnancy or during the first trimester (Table 1). A total of 103 case women (29.8%) reported exposure to housing renovations at home and/or at work. The *chi-square* test result for exposure to indoor renovations was 12.231 (*P* < 0.001) between cases and controls.

**Table 1:** An analysis of characteristics between the case and control groups

	**Controls**	**Cases**	***Chi square***	***P*-values**
	**N = 408 (%)**	**N = 346 (%)**		
Maternal age (yrs) ^a^				
< 20	8 (2.0)	10 (2.9)		
20 ~ 24	87 (21.3)	106 (30.6)	10.188	0.037*
25 ~ 29	193 (47.3)	145 (41.9)		
30 ~ 34	83 (20.3)	62 (17.9)		
≥35	37 (9.1)	23 (6.6)		
Maternal education level ^a^				
Primary school or less	1 (0.2)	12 (3.5)		
Junior school	53 (13.0)	104 (30.1)	54.685	<0.001*
Senior high school	106 (26.0)	86 (24.9)		
College or advance	242 (59.3)	134 (38.7)		
Missing	6 (1.5)	9 (2.6)		
Residence				
Urban	331 (81.1)	215 (62.1)		
Suburban	63 (15.4)	89 (25.7)	39.907	<0.001*
Rural	11 (2.7)	39 (11.3)		
Missing	3 (0.7)	3 (0.9)		
Housing renovation				
Yes	77 (18.9)	103 (29.8)	12.231	<0.001*
No	331 (81.1)	243 (70.2)		
Maternal smoking or exposure to ETS				
Yes	122 (29.9)	159 (46.0)	20.634	<0.001*
No	286 (70.1)	187 (54.0)		
Maternal cooking at home				
Yes	249 (61.0)	224 (64.7)	1.103	0.294
No	159 (39.0)	122 (35.3)		
Factory or landfill nearby				
Yes	75 (18.4)	100 (28.9)	11.624	0.001*
No	333 (81.6)	246 (71.1)		
Folic acid supplements				
Yes	368 (90.2)	272 (78.6)	19.575	<0.001*
No	40 (9.8)	74 (21.4)		
Indoor air ventilation ^a^				
Good	178 (43.6)	104 (30.1)	29.221	<0.001*
Average	215 (52.7)	203 (58.7)		
Poor	11 (2.7)	37 (10.7)		
Missing	4 (1.0)	2 (0.6)		
Maternal acute or chronic respiratory disease				
Yes	192 (47.1)	185 (53.5)	2.852	0.093
No	214 (52.5)	161 (46.5)		
Missing	2 (0.5)			

^a^ Baseline data were used in the following multivariate analysis as continuous variables.

* There were significant differences in proportions between the mothers from the case and control groups (tested two-tailed, *p* < 0.05).

### Effect analysis of indoor renovation on CHD occurrence

The effects of indoor renovation exposure on cases and controls are shown in Table 2. Mothers exposed to indoor renovations had an increased risk of giving birth to offspring with CHD (COR: 1.82; 95%CI: 1.30 to 2.56). After multivariate analysis with confounders, the risks for developing CHD was 1.89 (95%CI: 1.29 to 2.77). Furthermore, indoor renovations may be associated with a greater risk for the complex group (AOR: 2.65; 95%CI: 1.38 to 5.07) compared to the isolated cardiac defect group (AOR: 1.76; 95%CI: 1.18 to 2.64).

**Table 2:** Effect of indoor renovation exposure on groups of CHDs

	**Indoor renovation**		**COR (95%CI)**	**AOR**^**a **^**(95%CI)**
	**Yes**	**No**		
Controls	77	331	Reference	Reference
Cases	101	245	1.82 (1.30, 2.56)	1.89 ^**^ (1.29, 2.77)
CHD Groups				
Complex malformation	23	42	2.35 (1.34, 4.15)	2.65 ^**^ (1.38, 5.07)
Isolated Cardiac defect	80	201	1.71 (1.20, 2.45)	1.76^**^ (1.18, 2.64)
Subtype for isolated cardiac defect				
Septal defect	38	113	1.45 (0.93, 2.25)	1.56 (0.93, 2.62)
Conotruncal defect	34	102	1.43 (0.90, 2.27)	1.84 * (1.08, 3.13)
Right-sided obstructive	18	41	1.89 (1.03, 3.46)	1.41 (0.69, 2.88)
Left-sided obstructive	17	39	1.87 (1.01, 3.49)	1.51 (0.72, 3.17)
Anomalous venous return	12	23	2.24 (1.07, 4.70)	2.51 * (1.12, 5.66)
Others	15	30	2.15 (1.10, 4.19)	2.63 * (1.19, 5.80)

^a^ Variables entered were maternal age, maternal education level, place of residence (category), folate intake, ETS, factory or landfill nearby, air ventilation and hospital distribution (category).

Significant differences between the mothers of case and control groups were indicated as follows: * (p < 0.05), ** (p < 0.01), (tested two-tailed).

We further analyzed the effect of indoor renovation on subtypes of CHDs (Table 2). Among the offspring with isolated cardiac defects, exposure to housing renovations may have increased the risks to the fetuses who suffered from conotruncal defects, anomalous venous return and other types of CHD (*P* < 0.05). However, there was no significant influence on other CHD types, such as septal defects, right-side and left-side obstructive cardiac malformations.

### Effects of different time of renovation and living on CHD occurrence

The results of multivariate logistic analysis on different time of housing renovations and moving-in for the group with isolated cardiac defects are shown in Table 3. The highest risk of developing CHD for any group occurred when the interval time between renovation and moving-in was less than one month. An increased risk for giving birth to offspring with cardiac defects was found in only two groups where the mother moved into the decorated house either during 3 months before pregnancy (AOR: 2.38, 95%CI:1.03 to 5.48) or the first trimester (AOR: 4.00, 95%CI: 1.62 to 9.86), when the move-in occurred less than one month after renovation.

**Table 3:** Multivariate analysis of the time of housing renovations and moving-in on CHD occurrence

**Time of moving in renovated house**	**Interval between renovation and moving-in**	**Controls**	**Isolated cardiac defects**	***P*-value**	**AOR**^**a **^**(95%CI)**
		**N = 408 (%)**	**N = 281 (%)**		
No renovation		331 (81.1)	201 (71.5)		Reference
7-12 months before pregnancy	<1 months	3 (0.7)	3 (1.1)	0.664	1.50 (0.24, 9.37)
	1–3 months	3 (0.7)	1 (0.4)	0.992	0.99 (0.09, 11.04)
	≥4 months	2 (0.5)	3 (1.1)	0.256	3.01 (0.45, 20.15)
4-6 months before pregnancy	<1 months	3 (0.7)	6 (2.1)	0.108	3.59 (0.76, 17.04)
	1–3 months	5 (1.2)	4 (1.4)	0.727	1.33 (0.27, 6.62)
	≥4 months	4 (1.0)	3 (1.1)	0.659	1.42 (0.30, 6.85)
3 months before pregnancy	<1 months	13 (3.2)	18 (6.4)	0.042	2.38* (1.03, 5.48)
	1–3 months	8 (2.0)	8 (2.8)	0.346	1.70 (0.56, 5.16)
	≥4 months	12 (2.9)	3 (1.1)	0.222	0.42 (0.10, 1.69)
first trimester	<1 months	8 (2.0)	24 (8.5)	0.003	4.00** (1.62, 9.86)
	1–3 months	5 (1.0)	5 (1.8)	0.532	1.54 (0.40, 5.94)
	≥4 months	9 (2.2)	2 (0.7)	0.506	0.58 (0.12, 2.97)
Missing		2 (0.5)	0 (0.0)		

^a^ Variables entered were maternal age, maternal education level, place of residence (category), folate intake, ETS, factory or landfill nearby, air ventilation and hospital distribution (category).

Significant differences between the mothers of case and control group were indicated as follows: * (p < 0.05), ** (p < 0.01) (tested two-tailed).

## Discussion

The housing renovation activities in China have rapidly developed over the past two decades. Various kinds of renovation materials have been produced to improve the status of living. Housing decoration materials usually contain oil paints, dyes, laminate board, solid wood, marble, wallpaper, resin glue and plywood. A large number of environmental pollutants have been detected within these renovation materials. For example, organic solvents, heavy metals, and volatile organic compounds (VOCs), such as benzene, toluene, xylene, styrene and aldehyde, may be emitted from paints or dyes. Formaldehyde, trichloroethylene and VOCs can be found in boards or plywood. While radioactive substances such as radon may be emitted from marbles [17,24,25]. Large volumes of the contaminants may be released into the atmosphere during or after the indoor renovations. One Chinese study tested newly renovated houses and showed that only 20.75% of them met formaldehyde concentration level health standards. In addition, the passing rate was only 16.67% for VOCs in those houses [26]. Another study on the indoor detection showed that formaldehyde in as much as 80.96% of carpentry jobs and benzene in 35.71% of painting jobs were exceeded the standards for a renovation work environment [27]. Low-quality decoration materials may release much more pollutants into air; for example, excessive lead has been found in some brands of paints [9]. The indoor pollution phenomenon is widespread in China and some other countries [28,29]. Interior renovation contaminants, polluting indoor environments, are a new great threat to human health. However, there is a lack of evidence on the association between indoor renovation during the periconceptional time and the risk of adverse pregnancy outcomes, including CHD in offspring.

We found that maternal exposure to renovations may have an increased risk of giving birth to fetuses with CHD, which, to our knowledge, is the first view on housing renovation exposure showing an association with CHD in fetuses. This finding may be attributed to organic pollutants and other volatile contaminants being released from decoration materials. Maternal occupational exposure to organic solvents, such as Stoddard or Chlorinated solvents potentially increased the incidence rate of selected types of CHDs [30,31]. Some studies verified that trichloroethylene (TCE) can cause the developmental abnormalities in the hearts of avian embryos and mouse embryos [32,33]. In addition, epidemiological studies showed that exposure to benzene, TCE, and formaldehyde may increase the prevalence of CHD in offspring [32,34,35]. The increased risks for CHD occurrence from exposure to renovation activity may be due to those harmful substances being released from the decoration materials. Moreover, maternal exposure to organic dyes, lacquers, pigments and paints during the first trimester of pregnancy was found to be related to a higher incidence of cardiac malformations in fetuses [18,19]. Except for the occupational exposure, a DNBC study indicated that maternal non-occupational exposure to paint fumes may also be related to congenital abnormalities [20].

Because of the variety of confounding factors related to housing renovation, we first needed to determine the factors included in multivariate equations. We selected confounders based on the literature retrieval. Many studies have indicated that using folic acid before or during pregnancy may protect fetuses from some birth defects, including CHD [36,37]. A higher maternal education level and residence in a city have been shown to be protective elements for CHD [38]. Other factors such as maternal smoking or exposure to ETS [39,40], and factory or landfill nearby [40,41] are associated with the appearance of CHD in fetuses. In addition, the quality of air ventilation may also link to CHD. The structural characteristics, indoor temperature, absolute humidity and air-exchange rate of a building were shown to greatly affect the dynamic VOC emission rates [42] which may influence the concentration of air pollutants. The confounders with significant difference or contributing to the change of main effect were therefore recruited as covariate factors in the model.

Different phenotypes of CHDs showed different sensitivity to renovation exposure. Our result found that indoor renovations may increase the risk of the occurrence of conotruncal heart defects and anomalous venous return, which was confirmed by other studies. Just as the previous studies by Shaw and Tikkanen described earlier, mothers' occupational exposure to organic dyes (OR: 5.0) or pigments “end-use” (OR: 2.0) can increase the risk of conotruncal heart disease in offspring [19]. Exposure to certain chemicals like dyes, lacquers, pigments and paints, during the first trimester of pregnancy was found to be related to a higher incidence of conal malformations such as TGA, tetralogy of Fallot and truncus arteriosus [18,19]. Occupational exposure to Stoddard solvent may be associated with D-transposition of the great arteries (OR: 2.0) [31]. While benzene usage around the time conception/organogenesis increased the risk for neural crest malformations including double-outlet ventricle, Tetralogy of Fallot, and VSD (OR: 5.3; 95%CI: 1.4 to 21.1) [43]. Although we did not find an increased risk for septal defects and obstructions in our study, other research has shown maternal history of organic solvent exposure early in pregnancy may be associated with a slight increased risk for VSD (OR: 1.8) [44]; exposure was also associated with pure coarctation of the aorta (OR: 3.2) [45], aortic stenosis (OR: 2.1) and pulmonary valve stenosis (OR: 2.1) [31]. The different results may be due to sample sizes, different definitions of exposure, and different diagnostic abilities.

Our study showed a clear influence of time on the critical exposure windows of renovation activity for CHD occurrence. We can see that a shorter the interval between renovation and moving-in was associated with a greater risk that the offspring will suffer from CHD. Mothers who moved into a month-old redecorated house during the first trimester or 3 months before conception seemed to have an increased risk of giving birth to a baby with CHD. Similar results have not yet been reported in previous studies. Perhaps this finding can be explained by the dynamics of organic contaminants emission and elimination [46]. Heart is the first organ to form and function in the embryo. The first trimester is the critical window for heart development [47-49], so any risk factors that occur during this time may increase the risk of CHD. The concentration of VOCs released from the redecoration materials seemed to be the highest level in the newly renovated houses. The time at which VOCs from water-based paints, dry building materials and solvent-based paints are completely emitted is only approximately hundreds of seconds [42]. Over time, VOCs will slowly evaporate and the concentration will be gradually reduced [50,51]. In Howard’s test, the amount of formaldehyde emitted from conversion varnish coatings was 2.3-8.1 times higher than the amount of free formaldehyde applied [51]. The emission rate drops quickly in the first eight days, and then declines much more slowly over a longer period [51]. In addition, the elimination rates are strongly associated with air ventilation rates in houses [52].

In this article, we first reported the relationship between housing renovations and CHD occurrence. Maternal exposure to interior housing renovation activity may be associated with an increased risk of CHD in offspring. Moreover, different from the previous case–control study that investigated pregnant exposure during infancy or childhood period; our study started the epidemiologic investigation during pregnancy which may reduce errors in reporting exposure as much as possible. However, there were also some limitations to this study. First, like many epidemiological investigations, self-reported information from pregnant women would bring some bias. It is possible that case and control mothers may misclassify their exposure behaviors. In addition, a hospital-based study may introduce selection bias that influences the results. Therefore, further studies should be applied to test for the biomarkers on pollutions in biological samples of cases and controls in population. Second, due to a relatively small sample size, it was difficult to divide the CHD types and complexity factors into specific classifications. The number of cases was too small to have a high statistical power to assess the associations between renovations and CHD occurrence, especially for some selected phenotypes. A larger-scale prospective survey is needed in further investigations to enhance the efficacy of analysis. Finally, the relationship between renovation and indoor environmental pollution is complex. For example, new furniture is an important source of VOC, but this factor was not considered due to the low exposure rate. The types and qualities of renovation materials also have not been analyzed in this study. All of these problems should be considered in further studies.

## Conclusions

Maternal exposure to indoor renovation was associated with CHD occurrence in offspring with or without extra-cardiac defects. Living in a newly redecorated house may increase the fetus’ risk of selected types of cardiac defects. Mothers who moved into a renovated house less than one month after renovation during either the 3 months before conception or the first trimester have an increased risk of giving birth to offspring with CHD. The risk of housing renovation on CHD development needs to be clarified in further studies.

## Abbreviations

CHD: Congenital heart disease; AOR: Adjust odds ratio; COR: Crude odds ratio; 95% CI: 95% confidence interval; VOC: Volatile organic compound; ETS: Environmental tobacco smoke; VSD: Ventricular septal defects; TGA: Transposition of great arteries.

## Competing interests

The authors declare that they have no competing interests.

## Authors’ contributions

LZ and ZJ developed the study design, conducted the study, and drafted the manuscript; MD and WY assisted in organizing and implementing the project; LX performed data analysis and interpretation; LN, DK and DY participated in reviewing, editing, and revising the manuscript. LS, LY, CX, YF and LJ contributed to recruit participants and diagnosed the cases. All authors read and approved the final manuscript.
